# Multiple Eruptive Dermatofibromas in a Patient With HIV Infection: A Case Report

**DOI:** 10.7759/cureus.104664

**Published:** 2026-03-04

**Authors:** Lisa Tchifdjian, Ewa Hainaut, Rodolphe Riviere

**Affiliations:** 1 Dermatology, Poitiers University Hospital, Poitiers, FRA

**Keywords:** dermatofibromas, histiocytomas, hiv, human immunodeficiency virus, immunosuppression, multiple eruptive dermatofibromas

## Abstract

Multiple eruptive dermatofibromas represent a rare clinical entity characterized by the sudden development of numerous dermatofibromas. We report the case of a 49-year-old woman with a long-standing HIV infection who presented with the progressive development of multiple dermatofibromas over a three-year period. Multiple eruptive dermatofibromas are frequently associated with autoimmune diseases, malignancies, immunosuppressive treatments, or HIV infection. Recognition of this presentation is essential to avoid misdiagnosis and to prompt appropriate systemic evaluation.

## Introduction

Dermatofibromas, also known as histiocytomas, are common benign cutaneous tumors that typically present as solitary or a few lesions. The occurrence of multiple eruptive dermatofibromas is uncommon and is classically defined by the presence of more than 15 lesions in a single patient [1]. This condition has been reported in association with systemic diseases or immunosuppressive treatments. We report a case of multiple eruptive dermatofibromas occurring in a patient with HIV infection.

## Case presentation

**Figure 1:**
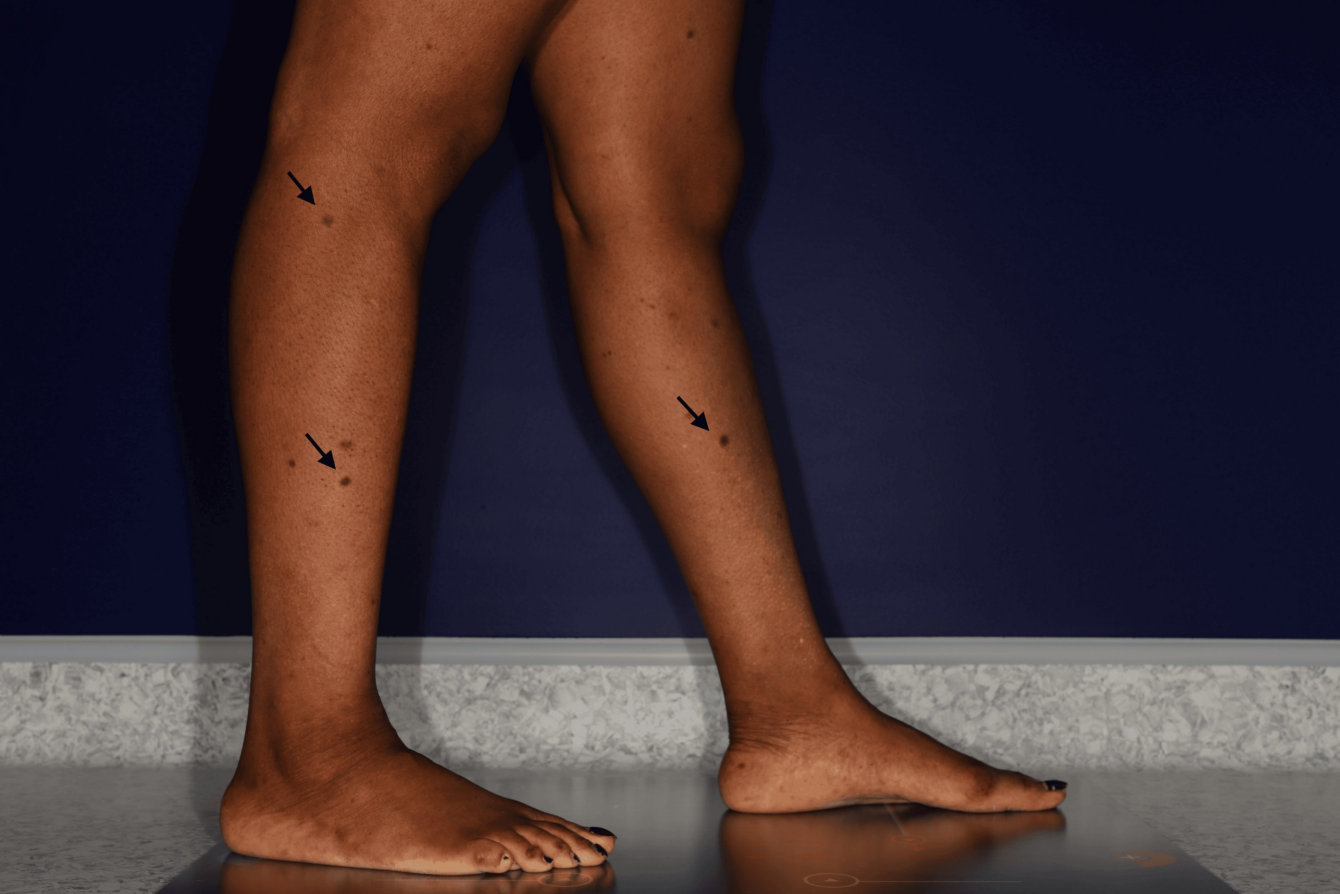
Multiple dermatofibromas on the lower limbs (indicated by black arrows)

A 49-year-old woman with a history of HIV infection diagnosed in 1997, complicated by *Pneumocystis jirovecii* pneumonia, was referred to the dermatology department in 2021 for the progressive appearance of multiple cutaneous lesions over a three-year period. Her antiretroviral therapy included dolutegravir, darunavir, and ritonavir. At the time of consultation, her viral load was undetectable. The patient reported the gradual development over three years of multiple pruritic, hyperpigmented nodular lesions (Figure 1).

**Figure 2:**
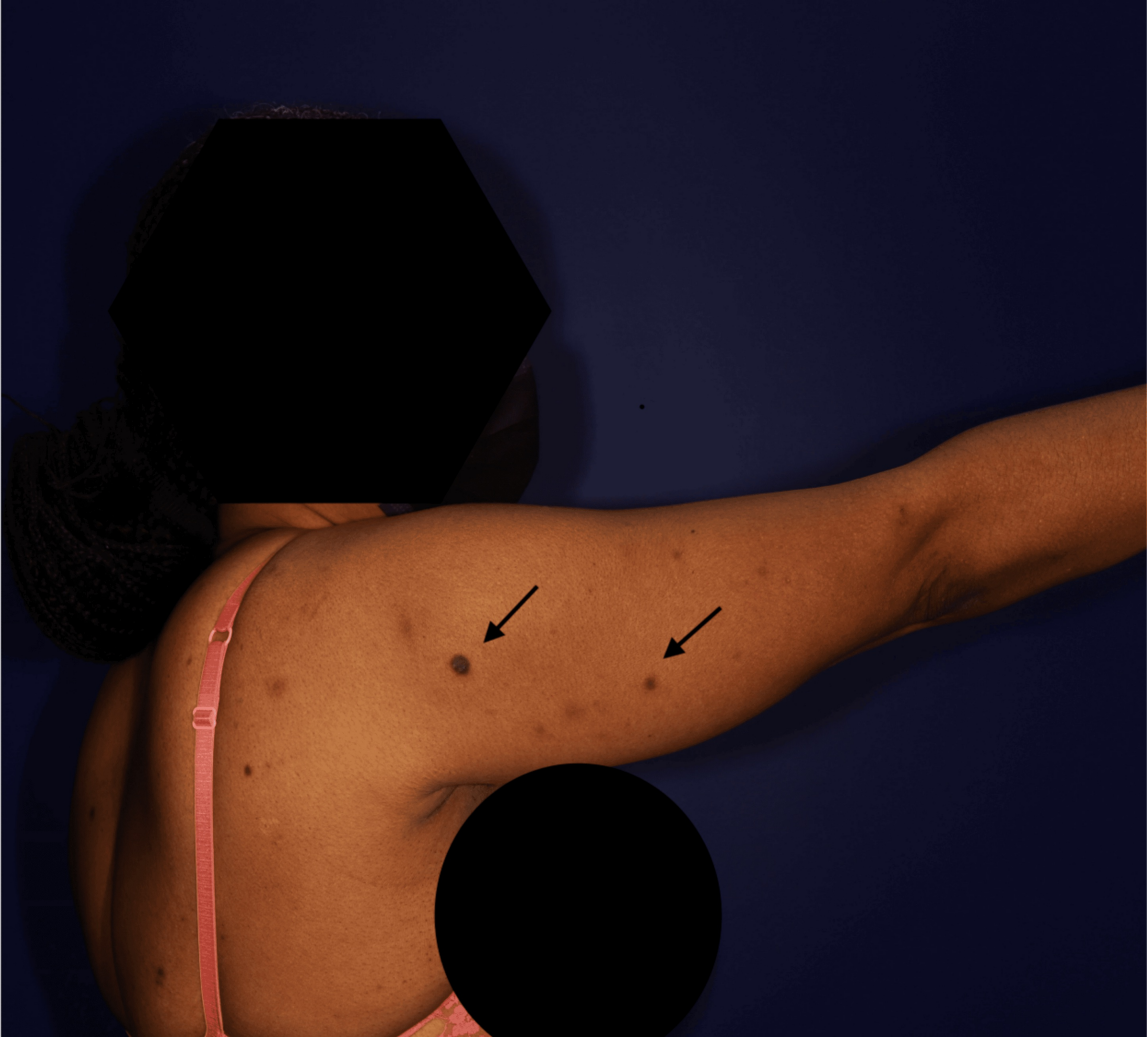
A close-up view of dark-brown firm papules on the back and arm (indicated by black arrows)

Clinical examination revealed 40 hyperpigmented papules and nodules, firm on palpation, distributed over the entire body, sparing the head (Figure 2). Dermoscopic examination showed homogeneous reticular pigmentation.

**Figure 3:**
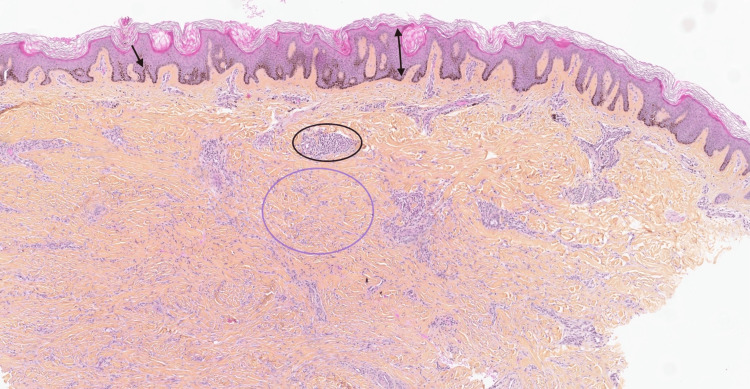
Biopsy of a dermatofibroma on the left arm (hematoxylin-eosin stain; original magnification: x 25) Histopathologic examination shows epidermal hyperplasia with elongation of rete ridges (double-headed arrow) and basal hyperpigmentation (arrow). The mid-dermis demonstrates a poorly circumscribed, storiform proliferation of bland spindle cells with entrapment of thickened collagen bundles (purple circled area). A mild perivascular and interstitial lymphocytic infiltrate is present (black circled area). No cytologic atypia or increased mitotic activity is identified.

The main differential diagnoses considered were multiple dermatofibromas, Kaposi sarcoma, granulomatous disease, and cutaneous leiomyoma. A skin biopsy from a lesion on the left arm revealed a poorly circumscribed dermal storiform proliferation of bland spindle cells with entrapment of thickened collagen bundles (Figure 3). No features of malignancy were identified. Immunohistochemical staining for human herpesvirus 8 (HHV-8) was negative, arguing against Kaposi sarcoma. These findings were consistent with dermatofibroma. 

Despite an undetectable viral load, HIV-related immunological dysregulation was considered the most likely contributing factor to the development of the lesions. Clinical and laboratory findings did not support systemic lupus erythematosus or other autoimmune diseases or malignancies. No therapeutic changes have been reported.

In the absence of functional impairment or cosmetic concerns, a conservative approach was adopted. The patient did not develop any new eruptive dermatofibromas during the following year and was subsequently lost to follow-up.

## Discussion

Approximately 100 cases of multiple eruptive dermatofibromas have been reported in the literature. This rare clinical entity is defined by the presence of more than 15 lesions in a single patient and predominantly affects women, accounting for approximately 61% of reported cases, with a mean age between 30 and 40 years [1].

Clinically, dermatofibromas display considerable morphological variability. Lesions may be papular or nodular, firm on palpation, and erythematous or hyperpigmented. Their size generally ranges from 0.5 to 1 cm. While most lesions are asymptomatic, some patients may report pruritus or pain. The lower limbs and trunk are the most commonly involved sites [1].

Although the diagnosis is often clinical, histopathological confirmation is recommended in order to exclude differential diagnoses such as Kaposi sarcoma, cutaneous leiomyoma, or granulomatous disorders. Like the more common presentation of solitary dermatofibromas, these lesions are benign, and therapeutic abstention remains appropriate [2].

In nearly two-thirds of cases, multiple eruptive dermatofibromas are associated with underlying systemic conditions, including autoimmune diseases, HIV infection, malignancies, or immunosuppressive therapies [1,3]. In a literature review conducted by Seifi et al. in 2022, approximately 11% of patients with multiple eruptive dermatofibromas had concomitant HIV infection [1].

In some patients, the appearance of multiple eruptive dermatofibromas has led to the diagnosis of HIV infection [2,4], while in others, lesions have developed following the initiation of effective antiretroviral therapy [4], possibly related to immune reconstitution. These observations support the hypothesis that immune dysregulation plays a central role in their pathogenesis.

In our patient, the multiple eruptive dermatofibromas were not associated with HIV disease progression, as the viral load remained undetectable, nor with any recent modification of antiretroviral therapy. The patient had no additional cutaneous lesions or systemic symptoms. No palpable lymphadenopathy was noted. Laboratory investigations, including complete blood count, serum protein electrophoresis, thyroid-stimulating hormone, and lactate dehydrogenase levels, were within normal limits. Consequently, no clear immunological trigger could be identified to explain the onset of the lesions.

The pathophysiology of multiple eruptive dermatofibromas remains poorly understood. Therefore, an appropriate etiological evaluation should be considered in patients presenting with multiple dermatofibromas, particularly in the absence of an obvious triggering factor.

## Conclusions

We report an additional case of multiple eruptive dermatofibromas associated with HIV infection. Although dermatofibromas are common and benign lesions, their multiple presentations are rare and may be associated with significant underlying systemic conditions. Recognition of this entity is important, as it should prompt clinicians to consider immunological or systemic evaluation.
